# Use of Sildenafil in Pulmonary Arterial Hypertension: Findings from a U.S. Healthcare Claims Database

**Published:** 2014-02-25

**Authors:** Ariel Berger, John Edelsberg, Simon Teal, Marko A. Mychaskiw, Gerry Oster

**Affiliations:** 1 Policy Analysis Inc. (PAI), Brookline, MA, USA; 2 Pfizer Ltd, Surrey, UK; 3 Pfizer, Inc., Collegeville, PA, USA

**Keywords:** medication adherence, pharmacotherapy, revatio®, sildenafil citrate, pulmonary arterial hypertension

## Abstract

**Background:** Pulmonary arterial hypertension (PAH) is a disease characterized by dyspnea, fatigue, chest pain and syncope. As there is no known cure for PAH, the goal of treatment is to control symptoms and slow disease progression. Sildenafil, a phosphodiesterase-5 inhibitor, has been indicated to improve exercise capacity in PAH in both the United States and the European Union since 2005; since 2009, it also has been indicated in the United States to delay clinical worsening. Patterns of sildenafil use in PAH patients have not been reported.

**Objectives:** To describe patterns of treatment with sildenafil among commercially insured patients in the United States with PAH.

**Methods:** Using a large U.S. healthcare claims database, we identified all patients with evidence of PAH (International Classification of Disease, 9th Revision, Clinical Modification [ICD-9-CM] diagnosis codes 416.0, 416.8) and receipt of sildenafil between January 1, 2005 and September 30, 2008. The date of each patient’s earliest pharmacy claim for sildenafil was designated as his or her “index date”; patients with <6 months of data prior to this date were excluded. Post-index use of sildenafil was then examined in terms of the numbers of pharmacy claims and therapy-days, the medication possession ratio (MPR), and the incidence of therapy switching.

**Results:** We identified a total of 855 PAH patients who began sildenafil therapy and met all other entry criteria. Mean (standard deviation [SD]) follow-up was 423.4 (313.0) days. Over this period, these patients averaged 7.1 (6.8) (median, 5) pharmacy dispensings for sildenafil, representing 273.4 (254.8) therapy-days (median, 180). Mean MPR was 71% (median, 83%). Fourteen percent of sildenafil patients switched to another agent during follow-up.

**Conclusions:** In “real-world” clinical practice, many PAH patients beginning treatment with sildenafil remain on therapy for extended periods and are relatively compliant with treatment.

## 1. Background

Pulmonary arterial hypertension (PAH) is a disease characterized by narrowing of the small arteries and arterioles, resulting in elevated pulmonary vascular resistance and increased pressure in the pulmonary arteries and leading eventually to right ventricular failure and death if untreated.[1,2] Symptoms of PAH include dyspnea, fatigue, chest pain and syncope.[3] Mean age at diagnosis is about 50 years, and most patients present initially with moderate-to-severe disease; about two-thirds of patients are women.[4]

PAH is one form of pulmonary hypertension, and is characterized hemodynamically as mean pulmonary arterial pressure (mPAP) of >25 mmHg with a mean pulmonary capillary wedge pressure (PWP) ≤15 mm Hg.[5] In the World Health Organization (WHO) classification of pulmonary hypertension, PAH constitutes Group 1, and includes both idiopathic PAH and PAH associated with other specific diseases.[6] In epidemiological studies, the most common types of PAH are (in order of decreasing frequency): (1) idiopathic PAH; (2) PAH associated with connective tissue disease; and (3) PAH associated with congenital systemic-to-pulmonary shunts in the heart.[4,7-9] Worldwide, it is estimated that between 130,000 and 260,000 persons have PAH.[9]

There is no known cure for PAH, and prognosis is poor; 5 year survival in the absence of treatment is only about 50%.[9,10] Currently, the goal of therapy is to control symptoms of the disease and hopefully slow its progress. Conventional therapy for PAH has included the management of underlying or contributing factors, avoidance of pregnancy, early treatment of respiratory tract infections, and immunization against pneumococcal disease and influenza.[11] Calcium channel blockers at high doses also have been an important component of conventional therapy in some PAH patients.[11]

In recent years, a number of targeted therapies have been introduced to treat PAH.[12] There are three main classes of such agents, which act on the three principal intracellular pathways: (1) prostaglandin/prostacyclin analogues (e.g., intravenous [IV] epoprostenol, nebulized or IV iloprost); (2) endothelin receptor antagonists (e.g., bosentan); and (3) phosphodiesterase-5 (PDE-5) inhibitors (e.g., sildenafil). All of these therapies have been shown to improve exercise capacity, hemodynamics, symptoms, and health-related quality of life.[13]

Sildenafil (Revatio®) is a PDE-5 inhibitor that has been indicated to improve exercise capacity in PAH in both the United States and the European Union since 2005; since 2009, it also has been indicated in the United States to delay clinical worsening.[14] Relatively little is known, however, about patterns of use of sildenafil in "real-world" settings. The objective of our study therefore was to describe sildenafil treatment patterns among commercially insured patients diagnosed with PAH in the United States.

## 2. Methods

### *Data Source*

Data were obtained from the Truven MarketScan Commercial Claims and Encounters Database, which consists of facility, professional-service, and retail (i.e., outpatient) pharmacy claims from a variety of private insurers that provide healthcare coverage to approximately 15 million persons annually throughout the United States. All patient identifiers in the database have been fully encrypted, and the database is fully compliant with the Health Insurance Portability and Accountability Act of 1996 (HIPAA) and federal guidance on Public Welfare and the Protection of Human Subjects; Institutional Review Board (IRB) review and approval was therefore not needed for this study.

Information available for each facility and professional-service claim includes date and place of service, diagnoses (in International Classification of Disease, 9th Revision, Clinical Modification [ICD-9-CM] format), procedures (in ICD-9-CM [selected plans only], Current Procedural Terminology [CPT]-4, and Healthcare Common Procedure Coding System [HCPCS] formats), provider specialty, and charged and paid amounts. Data available for each retail pharmacy claim include the drug dispensed (in National Drug Code [NDC] format), the dispensing date, and the quantity dispensed and number of days of therapy supplied (selected plans only). All claims include a charged amount; the database also provides paid (i.e., reimbursed, including patient deductible, copayment and/or coinsurance) amounts.

Selected demographic and eligibility information is also available, including age, gender, geographic region, coverage type and dates of insurance coverage. All patient-level data can be arrayed chronologically to provide a detailed, longitudinal profile of all medical and pharmacy services used by each plan member. The database for this study encompassed the period, January 1, 2005 through September 30, 2008 ("study period").

*Study Sample*

The source population consisted of all persons with any inpatient claims, or two or more outpatient claims at least 30 days apart, with ICD-9-CM diagnosis codes consistent with PAH (416.0, 416.8) between January 1, 2005 and September 30, 2008. We used diagnoses for both primary (416.0) and secondary (416.8) PAH in order to completely capture all patients with PAH. Among these patients, we then identified those with any evidence of receipt of (i.e., any pharmacy claims for) Revatio®, the formulation of sildenafil that is indicated for PAH (another formulation of sildenafil is indicated for the treatment of erectile dysfunction and is sold under the brand name Viagra®; the dosages of Revatio® and Viagra® differ, however, as do the number of pills typically supplied with each prescription). The date of each patient's first claim for sildenafil was designated as his or her "index date". Patients with <6 months of enrollment data prior to their index date ("pre-index period") were excluded, as were those aged <18 years (on index date), and those aged ≥65 years (on index date) if they were not enrolled in a Medicare risk-sharing plan (the claims histories of patients aged ≥65 years in other types of health plans may be incomplete in the database). All available information was then compiled for these patients, beginning 6 months prior to their index date and ending with the end of study period (or date of health plan disenrollment, irrespective of reason, if earlier).

*Measures and Analysis*

The demographic and clinical characteristics of study subjects, including prevalence of selected comorbidities, were characterized on the basis of information during the 6-month pre-index period. Patients were designated as having a particular comorbidity if they had any hospitalizations, or two or more outpatient claims at least 30 days apart, during the pre-index period with a corresponding diagnosis code/prescription (Table 1). The Charlson Comorbidity Index score was also calculated.[15]

We also examined levels of healthcare utilization and cost during the 6-month pre-index period with particular focus on medications related to the treatment of PAH (PDE-5 inhibitors [other than sildenafil], prostaglandin/prostacyclin analogues, endothelin receptor antagonists, nitric oxide/nitric oxide donors, calcium channel blockers (CCBs), oral anticoagulants/antiplatelets, diuretics, oxygen, and cardiac glycosides) (collectively, "PAH-related therapies"). We also expressed each patient's total cost of PAH-related care during the pre-index period as a proportion of the mean cost of PAH-related care for all patients in the study sample; we used this measure of intensity of care as a rough proxy for each patient's disease severity.

**Table 1. Definitions for Comorbidities of Interest**

| Comorbidity | Definition |
|---|---|
| **Connective tissue diseases** | ICD-9-CM diagnoses 710.0, 710.1, 714.X, 710.3, 710.4 |
| **Congenital heart diseases** | ICD-9-CM diagnoses 745.3, 745.4, 745.5, 745.6, 747.0 |
| **HIV/AIDS** | ICD-9-CM diagnoses 042, 079.53, V08 |
| **Depressive disorders** | ICD-9-CM diagnoses 311, 296.2X, 296.3X, 296.5X, 296.82, 300.4, 298.0, 309.0, 309.28, 309.1 |
| **Anxiety disorders** | ICD-9-CM diagnoses 300.XX, 301.XX, 309.21 |
| **Sleep disorders** | ICD-9-CM diagnoses 780.57, 780.50, 780.51, 780.53, 307.4X, 780.5X, V69.4 |
| **Lung disease** | |
| **Asthma** | ICD-9-CM diagnoses 493.XX |
| **Chronic obstructive pulmonary disease** | ICD-9-CM diagnoses 491.XX, 492.XX, 496.XX |
| **Pulmonary fibrosis** | ICD-9-CM diagnoses 515, 516.3 |
| **Cerebrovascular disease** | ICD-9-CM diagnoses 430-438.XX |
| **Coronary heart disease** | ICD-9-CM diagnoses 410-414.XX |
| **Atrial fibrillation** | ICD-9-CM diagnoses 427.3 |
| **Congestive heart failure** | ICD-9-CM diagnoses 428.XX |
| **Peripheral vascular disease** | ICD-9-CM diagnoses 440.2X, 440.3X, 443.9X, 444.22 |
| **Hemtaological conditions** | ICD-9-CM diagnoses 282.6, 282.49, 289.6, 205.1X |
| **Liver disease** | ICD-9-CM diagnoses 572.3, 006.3, 070.22, 070.32, 070.33, 070.44, 070.54, 456.0-456.21, 570-572.29, 572.4-573.9, 996.82, V42.7 |
| **Renal disease** | ICD-9-CM diagnoses 293.9, 294.8, 276.0-276.9, 458.21, 567.XX, 584.XX-586.XX, 792.5, 996.1, 996.62, 996.56, 996.68, 996.73, 999.2, 999.3, 999.9, V45.1, V56.0, V56.1, V56.2, V56.3X, V56.8, E87.02, E87.91; ICD-9-CM procedures 39.42, 39.43, 39.93, 39.94, 39.9 |
| **Diabetes** | ICD-9-CM diagnoses 250.XX; receipt of alpha-glucosidase inhibitors, insulin, metformin, nonsulfonylurea insulin secretagogues, sulfonylurea, or thiazolidinedione |
| **Neoplasms** | ICD-9-CM diagnosis 140.XX-209.XX, 230.XX-238.XX |

ICD-9-CM: International Classification of Disease, 9th Revision, Clinical Modification

Post-index use of sildenafil was examined in terms of the numbers of pharmacy claims and therapy-days. Adherence with sildenafil was examined using medication possession ratios (MPRs) and cumulative medications gaps (CMGs).[16] MPR was calculated as the ratio of the total number of therapy-days supplied during follow-up (beginning on the index date, and ending with last day of follow-up) to the total number of calendar-days of follow-up. Therapy-days that extended beyond the last day of follow-up were truncated as of the last day of follow-up, and days in hospital were subtracted from total days of follow-up for purposes of calculating MPR. CMG was calculated as the ratio of the difference between the total number of days of follow-up and the total number of covered days, to the total number of days of follow-up. Therapy switching also was examined; in these analyses, patients were assumed to have switched therapy if: (1) they had any evidence of receipt of a PAH-related therapy other than sildenafil (including the alternative preparation of sildenafil [i.e., Viagra®]) during follow-up that they did not receive during the pre-index period; and (2) they had no evidence of receipt of sildenafil ≥30 days following the date of first receipt of a new PAH-related therapy. The therapy switching date was assumed to be the date of first receipt of the new PAH-related therapy.

All analyses were conducted using PC-SAS® v.8.4 (SAS, Cary NC).

## 3. Results

We identified a total of 855 patients who began sildenafil therapy, had evidence of PAH, and met all other entry criteria (Table 2). Most (86.0%) patients had medical encounters with diagnoses of primary and secondary pulmonary hypertension; 4.3% had diagnoses of primary pulmonary hypertension only, and 9.7% had diagnoses of secondary pulmonary hypertension only. Mean (standard deviation [SD]) age was 52.6 (9.9) years; 69.4% were women (Table 3). Thirty-two percent of patients had comorbid lung disease (primarily, chronic obstructive pulmonary disorder [COPD] [20.0% of all study subjects]); 27.3% had congestive heart failure (CHF); 17.4%, connective tissue disease; 17.2%, diabetes; and 15.9%, renal disease. Mean and median (interquartile range [IQR]) total healthcare costs during the pre-index period were $42,166 ($65,256) and $24,484 ($12,417, $43,680), respectively.

**Table 2. Sample Selection**

| Criteria | Number of Patients |
|---|---|
| Total number of patients with ≥1 inpatient claims, or ≥2 outpatient claims at least 30 days apart, with diagnosis of PAH during study period, and | 22,101 |
| One or more pharmacy claims for sildenafil[b] during study period and | 1,116 |
| ≥6 months enrollment prior to index date and | 899 |
| Eligible for medical and pharmacy benefits for duration of study period and | 899 |
| Aged ≥18 years as of index date and | 856 |
| Aged <65 years as of index date or | 856 |
| Aged ≥65 years as of index date and enrolled in Medicare risk-sharing plan and | 856 |
| Total costs ≥$0 | 855 |
| Total in study sample | 855 |

[a]Spanning January 1, 2005 to end of database; [b]Revatio® preparation; PAH: Pulmonary arterial hypertension

During the 6-month pre-index period, 64.8% of study subjects received diuretics; 35.9%, calcium channel blocker; 34.9%, oral anticoagulants; and 27.3%, endothelin receptor antagonists (Figure 1); 74.3% received medications from two or more of the above-listed classes. During this period, they also averaged 0.2 (0.8) pharmacy claims for phosphodiesterase type-5 inhibitors—primarily Viagra®—representing 4.6 (22.4) therapy-days; 1.2 (2.2) pharmacy claims for endothelin receptor antagonists, representing 32.8 (59.3) therapy-days; 1.1 (1.9) pharmacy claims for CCBs, representing 37.5 (59.4) therapy-days; 1.1 (1.9) pharmacy claims for oral anticoagulants, representing 33.1 (55.3) therapy-days; 3.1 (3.8) pharmacy claims for diuretics representing 67.7 (64.4) therapy-days and 0.5 (1.3) pharmacy claims for cardiac glycosides (exclusively digoxin) representing 16.0 (42.7) therapy-days (Table 4). Study subjects also averaged 3.2 (4.6) claims for oxygen an average of 26.2 (12.6) days apart, and 0.3 (1.3) claims for epoprostenol an average of 26.6 (11.4) days apart.

Mean (SD) duration of follow-up was 423.4 (313.0) days; median (IQR) follow-up was 357 (160, 643) days. During follow-up, patients averaged 7.1 (6.8) pharmacy claims for sildenafil, representing 273.4 (254.8) therapy-days; corresponding median (IQR) values were 5 (2, 10) and 180 (70, 412), respectively (Table 5). The frequency distribution of therapy-days with sildenafil is shown in Figure 2. Although the course of therapy was relatively short (≤90 days) for approximately one-third of patients, 19.7% received 181 to 365 days of therapy, 21.3% received 366 to 730 days of therapy, and 7.6% received >730 days of therapy. Mean (SD) and median (IQR) MPR were 71.1% (30.9%) and 83% (49%, 98%), respectively; corresponding values for CMG were 28.9% (30.9%) and 17% (2%, 51%). Fourteen percent of patients switched to a new PAH-related therapy during follow-up (Figure 3). Among those who switched to a new medication, approximately one-quarter did so within 6 months of beginning sildenafil therapy; approximately one-half, within 1 year of beginning therapy; and approximately 80%, within 2 years of beginning therapy.

**Table 3. Demographic and Clinical Characteristics of Study Subjects in the Full Dataset (N=855)**

| Characteristic | Value | |
|---|---|---|
| Mean (SD) age, y | 52.6 | (9.9) |
| Number (%) women | 593 | (69.4) |
| Comorbidities, n (%) | | |
| Connective tissue diseases | 149 | (17.4) |
| Congenital heart diseases | 35 | (4.1) |
| HIV/AIDS | 3 | (0.4) |
| Depressive disorders | 41 | (4.8) |
| Anxiety disorders | 17 | (2.0) |
| Sleep disorders | 120 | (14.0) |
| Lung disease | | |
| Asthma | 57 | (6.7) |
| Chronic obstructive pulmonary disease | 171 | (20.0) |
| Pulmonary fibrosis | 104 | (12.2) |
| Any of above | 272 | (31.8) |
| Cerebrovascular disease | 26 | (3.0) |
| Coronary heart disease | 131 | (15.3) |
| Atrial fibrillation | 76 | (8.9) |
| Congestive heart failure | 233 | (27.3) |
| Peripheral vascular disease | 19 | (2.2) |
| Hemtaological conditions | 10 | (1.2) |
| Liver disease | 75 | (8.8) |
| Renal disease | 136 | (15.9) |
| Diabetes | 147 | (17.2) |
| Neoplasms | 64 | (7.5) |
| Care intensity index for PAH-related services during pre-index period | | |
| <1.0 | 628 | (73.5) |
| 1.0-1.9 | 119 | (13.9) |
| 2.0-2.9 | 44 | (5.1) |
| ≥3.0 | 64 | (7.5) |
| Total healthcare costs during pre-index period, $ | | |
| Mean (SD) | 42,166 | (65,256) |
| Median (IQR) | 24,484 | (12,417, 43,680) |
| Geographic residence (n [%]) | | |
| Northeast | 76 | (8.9) |
| North central | 231 | (27.0) |
| South | 428 | (50.1) |
| West | 114 | (13.3) |
| Unknown/missing | 6 | (0.7) |
| Payer type (n [%]) | | |
| HMO | 127 | (14.9) |
| POS | 98 | (11.5) |
| PPO | 485 | (56.7) |
| Other/unknown | 145 | (17.0) |

SD: standard deviation; PAH: Pulmonary arterial hypertension; HMO: Health maintenance organization; PPO: Preferred provider organization; POS: Point of service

**Figure 1. Percentage of Study Subjects receiving PAH-related Therapies during the Pre-index Period**

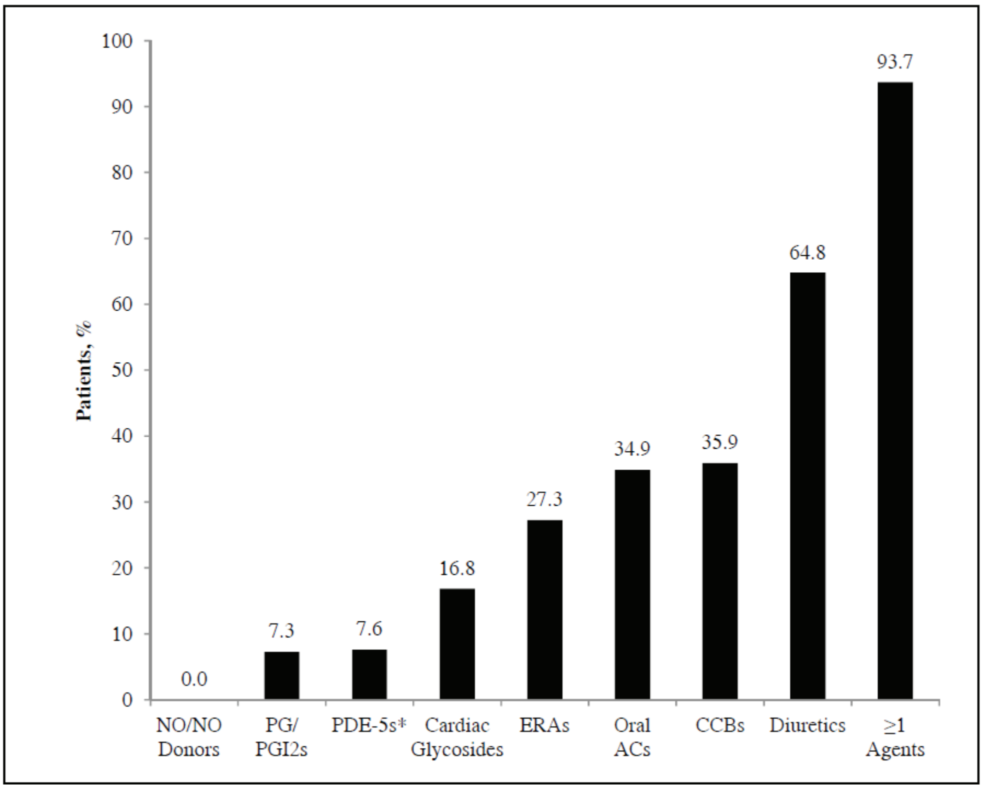

*Including Viagra®; NO: nitric oxide; PG/PGI2: prostaglandin/prostacyclin analogue; PDE-5: phosphodiesterase type-5 inhibitor; ERA: endothelin receptor antagonist; AC: anticoagulant; CCB: calcium channel blocker

**Table 4. Magnitude of Use of PAH-related Therapies during Pre-index Period**

| PAH-related Therapy | Prescriptions | | Therapy-Days | |
|---|---|---|---|---|
| Phosphodiesterase type-5 inhibitors[a] | | | | |
| Mean (SD) | 0.2 | (0.8) | 4.6 | (22.4) |
| Median (IQR) | 0 | (0, 0) | 0 | (0, 0) |
| Prostaglandin/prostacyclin analogues | | | | |
| Mean (SD) | 0.3 | (1.5) | 7.8 | (32.5) |
| Median (IQR) | 0 | (0, 0) | 0 | (0, 0) |
| Endothelin receptor antagonists | | | | |
| Mean (SD) | 1.2 | (2.2) | 32.8 | (59.3) |
| Median (IQR) | 0 | (0, 2) | 0 | (0, 30) |
| Nitric oxide/nitric oxide donors | | | | |
| Mean (SD) | 0.0 | (0.0) | 0.0 | (0.0) |
| Median (IQR) | 0 | (0, 0) | 0 | (0, 0) |
| Calcium channel blockers | | | | |
| Mean (SD) | 1.1 | (1.9) | 37.5 | (59.4) |
| Median (IQR) | 0 | (0, 2) | 0 | (0, 85) |
| Oral anticoagulants | | | | |
| Mean (SD) | 1.1 | (1.9) | 33.1 | (55.3) |
| Median (IQR) | 0 | (0, 2) | 0 | (0, 60) |
| Diuretics | | | | |
| Mean (SD) | 3.1 | (3.8) | 67.7 | (64.4) |
| Median (IQR) | 2 | (0, 5) | 60 | (0, 128) |
| Cardiac glycosides | | | | |
| Mean (SD) | 0.5 | (1.3) | 16.0 | (42.7) |
| Median (IQR) | 0 | (0, 0) | 0 | (0, 0) |
| All of above | | | | |
| Mean (SD) | 10.8 | (8.5) | 127.5 | (56.4) |
| Median (IQR) | 9 | (5, 15) | 154 | (99, 170) |

[a]Including Viagra®; PAH: Pulmonary-arterial hypertension; SD: standard deviation; IQR: interquartile range

**Table 5. Magnitude of Sildenafil Use during the Follow-up Period[a]**

| | Value | |
|---|---|---|
| **Prescriptions** | | |
| Mean (SD) | 7.1 | (6.8) |
| Median (IQR) | 5 | (2, 10) |
| Minimum | 1 | |
| Maximum | 43 | |
| **Therapy-days** | | |
| Mean (SD) | 273.4 | (254.8) |
| Median (IQR) | 180 | (70, 412) |
| Minimum | 1 | |
| Maximum | 1,146 | |
| **MPR** | | |
| <10% | 45 | (5.3) |
| 10-20% | 51 | (6.0) |
| 21-30% | 47 | (5.6) |
| 31-40% | 30 | (3.5) |
| 41-50% | 47 | (5.6) |
| 51-60% | 44 | (5.2) |
| 61-70% | 50 | (5.9) |
| 71-80% | 82 | (9.7) |
| 81-90% | 98 | (11.6) |
| 91-100% | 352 | (41.6) |
| Mean (SD) | 71.1 | (30.9) |
| Median (IQR) | 83 | (49, 98) |
| Minimum | 2 | |
| Maximum | 100 | |
| **CMG** | | |
| <10% | 353 | (41.7) |
| 10-20% | 100 | (11.8) |
| 21-30% | 80 | (9.5) |
| 31-40% | 49 | (5.8) |
| 41-50% | 44 | (5.2) |
| 51-60% | 47 | (5.6) |
| 61-70% | 32 | (3.8) |
| 71-80% | 45 | (5.3) |
| 81-90% | 51 | (6.0) |
| 91-100% | 45 | (5.3) |
| Mean (SD) | 28.9 | (30.9) |
| Median (IQR) | 17 | (2, 51) |
| Minimum | 0 | |
| Maximum | 98 | |

[a]Unless otherwise indicated, all values are number of patients (%); Note: 9 patients were excluded from these analyses due to missing values for therapy-days; MPR: Medication possession ratios; CMG: Cumulative medication gap; SD: standard deviation; IQR: interquartile range

**Figure 2. Frequency Distribution of Sildenafil Therapy-days among Study Subjects Newly Started on such Therapy**

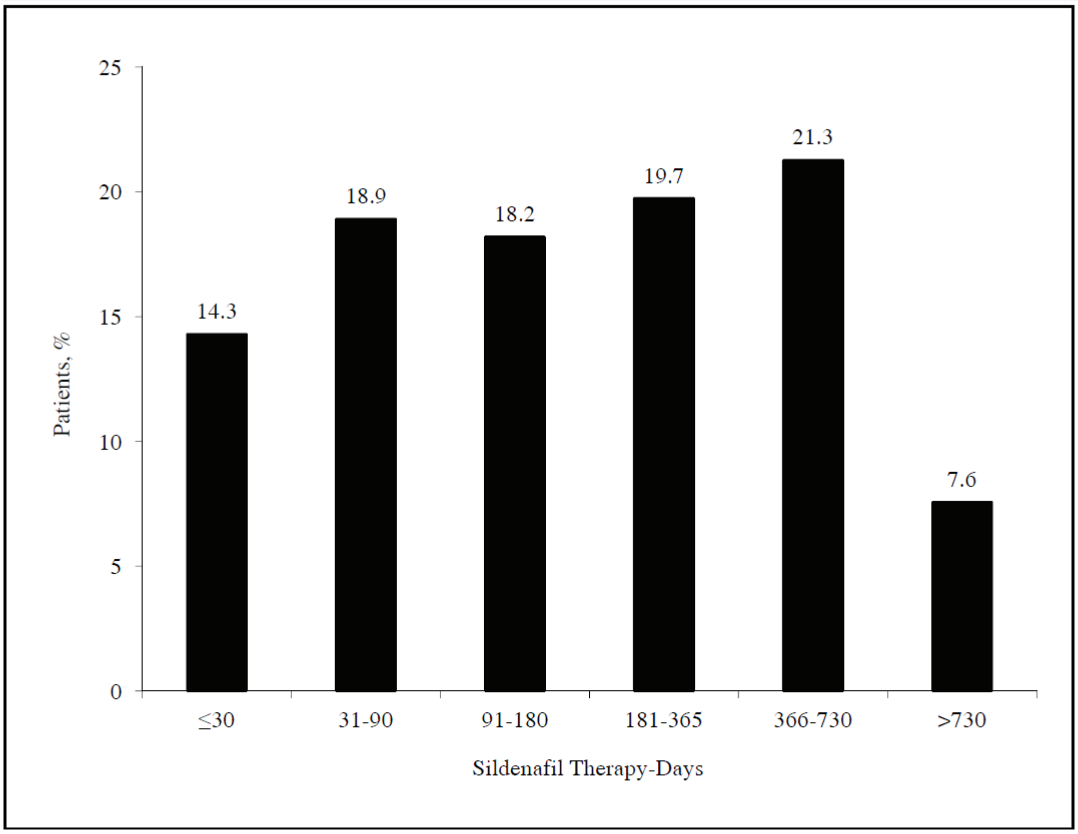

**Figure 3. Incidence of Therapy Switching from Sildenafil among Study Subjects during the Follow-up Period**

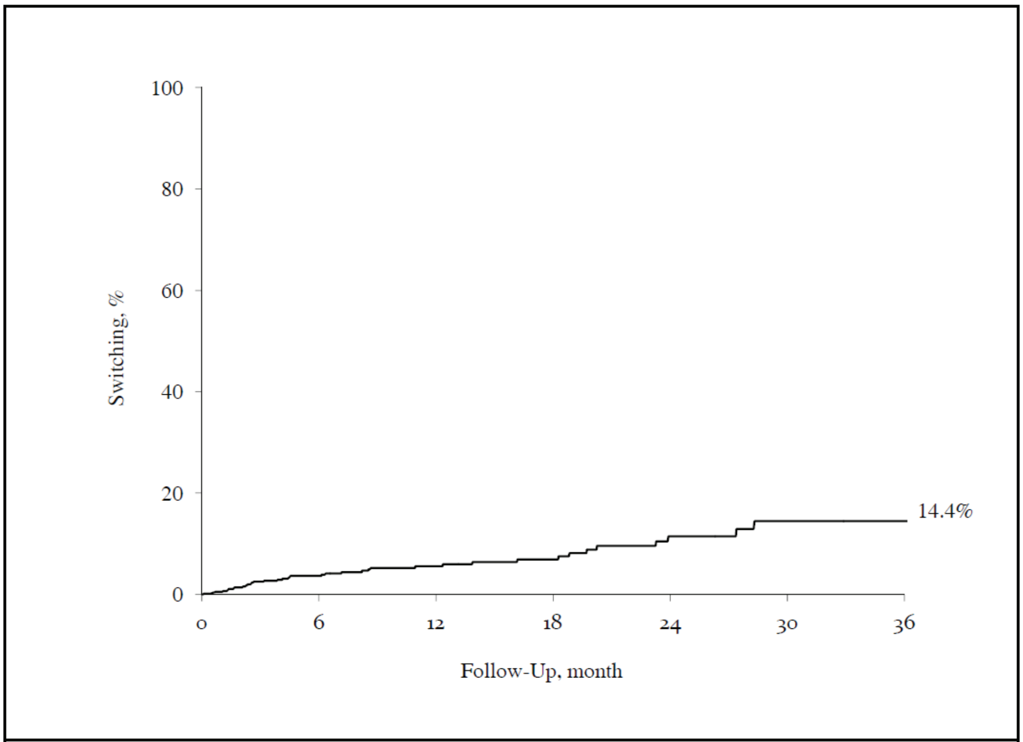

| | **0** | **6** | **12** | **18** | **24** | **30** | **36** |
|---|---|---|---|---|---|---|---|
| | | | | **Number at Risk** | | | |
| Study subjects | 855 | 417 | 251 | 160 | 91 | 38 | 6 |

## 4. Discussion

Almost one-half of PAH patients beginning treatment with sildenafil received courses of therapy lasting more than six months, and 29% received courses of therapy lasting more than 1 year. Only 14% of patients switched to a new PAH-related therapy, and overall compliance was quite high. In studies of other drugs, MPRs ≥80% are usually considered to indicate good compliance with pharmacotherapy;[17-20] mean and median MPRs in our study were 71.3% and 83.3%, respectively.

Unfortunately, measures of clinical efficacy (e.g., the 6-minute walk test) were not available in our database. We therefore have no direct evidence of the effectiveness of sildenafil in our study population. How, then, should our findings be interpreted? Factors known to foster poor compliance include side effects, patients' lack of belief in the benefits of treatment, psychological problems (especially depression), and high cost of medication, copayment, or both.[21] Against the backdrop of prior research, we think that it is reasonable to assume that the substantial numbers of study subjects with long-term use of sildenafil, coupled with high MPRs, suggest good tolerability and at least a reasonable degree of effectiveness when prescribed for PAH.

The principal objective of our study was to characterize patterns of utilization of sildenafil for PAH. Our analyses therefore were exclusively descriptive in nature. While our findings have shed light on how sildenafil is actually used in the United States in patients with PAH, other important questions remain unanswered. We do not know, for example, whether patterns of utilization—including therapy compliance, persistency, and switching—differ in clinical practice between sildenafil and other medications used to treat PAH, such as bosentan and epoprostenol. As this was beyond the scope of our study, we hope that further research will examine these questions, using techniques, such as propensity score matching, that can provide a basis for unbiased comparisons between treatment groups.

Our study has several notable limitations inherent in studies based on U.S. healthcare claims data. First, study patients were considered to have PAH based on one or more inpatient claims, or two or more outpatient claims at least 30 days apart, with either of two ICD-9-CM diagnosis codes (416.0 or 416.8). Whether they would have met clinical criteria for PAH is not ascertainable, since clinical data such as cardiopulmonary hemodynamics are not available in claims databases. On a related note, while 90% of study patients had at least one claim for ICD-9-CM 416.0 (primary pulmonary hypertension, which is specific for PAH if used appropriately), 86% had claims for both ICD-9-CM 416.0 and 416.8 (the latter, a non-specific code that is described as "other chronic pulmonary heart disease" (i.e., pulmonary hypertension groups 2-4). Twenty-seven percent of study subjects had CHF (a common underlying condition in Group 2 pulmonary hypertension), and 20% had COPD (a common underlying condition in Group 3 pulmonary hypertension). While it is possible to have both PAH and COPD and/or CHF (right-heart failure develops late in the course of PAH), the actual number of patients with PAH and COPD/CHF is likely quite small. It is more likely that many of the patients with CHF/COPD had Group 2 or Group 3 pulmonary hypertension, and thus the 416.0 code was inappropriately used. On the other hand, even if patients with CHF and COPD represent two mutually exclusive subgroups (i.e., no patient had both CHF and COPD), this would account for only about one-half of all patients with both diagnosis codes for pulmonary hypertension. Moreover, because all study patients received sildenafil, which is approved only for PAH and not for other forms of pulmonary hypertension, we believe that the proportion of study patients with non-PAH pulmonary hypertension (i.e., those patients with group 2-4 disease who were prescribed sildenafil off-label) was relatively small. While physicians in clinical practice may prescribe drugs approved for PAH for their patients with secondary pulmonary hypertension, since sildenafil has not been proven efficacious for other groups of pulmonary hypertension, their inclusion would impart a conservative bias to our findings (i.e., as a result of presumed early discontinuation of sildenafil due lack of efficacy in these patients). Given that patients' medical records were unavailable, the degree to which such misclassification impacts our sample is unknowable.

Second, healthcare claims databases only allow for the identification of prescription drugs dispensed by retail pharmacies (i.e., filled prescriptions and their associated therapy-days). We could not ascertain whether medications that were dispensed were actually taken. Thus, our estimates may represent an upper bound of the amount of sildenafil—and all other PAH-related therapies for that matter—that was actually used. Given the relatively high MPRs noted in our study, however, we believe it is reasonable to assume that most patients who filled prescriptions for sildenafil actually adhered with such therapy.

Finally, as with all studies of healthcare claims data, there may be errors of omission and commission in coding; without access to patients' medical records, however, the degree to which this occurred in claims used in our study—and the resulting impact of any such misclassification on our findings—is unknown.

## 5. Conclusion

In "real-world" clinical practice, many PAH patients beginning treatment with sildenafil remain on therapy for extended periods and are relatively compliant with treatment.

## Conflict of Interest Declaration

The study was sponsored by Pfizer, Inc., New York, NY, USA. AB, JE, and GO are employees of Policy Analysis Inc. (PAI), who were paid consultants to Pfizer, Inc. in the development of this manuscript. ST and MM are employees of Pfizer, Inc. All work was undertaken at PAI, Brookline, MA.

## References

1 Galie N, Hoeper MM, Humbert M, *et al*: **Guidelines for the diagnosis and treatment of pulmonary hypertension: The Task Force for the Diagnosis and Treatment of Pulmonary Hypertension of the European Society of Cardiology (ESC) and the European Respiratory Society (ERS), endorsed by the International Society of Heart and Lung Transplantation (ISHLT).** *Eur Heart J* 2009;**30**(20):2493-537.

2 Channick R, Williamson TL: **Diagnosis and treatment of pulmonary arterial hypertension.** *Cardiol Clin* 2004;**22**(3):441-52.

3 Rich S, Dnatzker DR, Ayres SM, *et al:* **Primary pulmonary hypertension. A national prospective study.** *Ann Intern Med* 1987;**107**(2):216-23.

4 Humbert M, Sitbon O, Chaouat A, *et al:* **Pulmonary arterial hypertension in France: Results from a national registry.** *Am J Respir Crit Care Med* 2006;**173**(9):1023-30.

5 Badesch DB, Champion HC, Sanchez MA, *et al:* **Diagnosis and assessment of pulmonary arterial hypertension.** *J Am Coll Cardiol* 2009;**54**(1 Suppl):S55-66.

6 Simonneau G, Robbins IM, Beghetti M, *et al:* **Updated clinical classification of pulmonary hypertension.** *J Am Coll Cardiol* 2009;**54**(1 Suppl):S43-54.

7 Peacock AJ, Murphy NF, McMurray JJ, *et al:* **An epidemiologic study of pulmonary arterial hypertension.** *Eur Respir J* 2007;**30**(1):104-9.

[8] Thenappan T, Shah SJ, Rich S, *et al:* **A USA-based registry for pulmonary arterial hypertension: 1982-2006.** *Eur Respir J* 2007;**30**(6):1103-10.

[9] D'Alonzo GE, Barst RJ, Ayres SM, *et al:* **Survival in patients with primary pulmonary hypertension: Results from a national prospective registry.** *Ann Intern Med* 1991;**115**(5):343-9.

[10] National Library of Medicine Medical Encyclopedia. **Pulmonary Hypertension.** http://www.nlm.gov/medlineplus/ency/article/000112.htm. Accessed March 13, 2012.

[11] Barst RJ, Gibbs JS, Ghofrani HA, *et al:* **Updated evidence-based treatment algorithm in pulmonary arterial hypertension.** *J Am Coll Cardiol* 2009;**54**(1 Suppl):S78-84.

[12] Hamilton N: Therapy Review: **An independent review of the currently available data for treatment of hypertension in the UK.** Pulmonary Hypertension Association (UK), 2006.

[13] Galie N, Manes A, Negro L, *et al:* **A meta-analysis of randomized controlled trials in pulmonary arterial hypertension.** *Eur Heart J* 2009;**30**(4):394-403.

[14] **Revatio™ prescribing information.** http://media.pfizer.com/files/products/uspi_revatio.pdf. Accessed March 15, 2012.

[15] Deyo RA, Cherkin DC, Ciol MA: **Adapting a clinical comorbidity index for use with ICD-9-CM administrative databases.** *J Clin Epidemiol* 1992;**45**(6):613-9.

[16] Cramer JA, Roy A, Burrell A, *et al:* **Medication compliance and persistence: Terminology and definitions.** *Value Health* 2008;**11**(1):44-7.

[17] Sikka R, Xia F, Aubert RE: **Estimating medication adherence using administrative claims data.** *Am J Manag Care* 2005;**11**(7):449-57.

[18] Rizzo JA, Simons WR: **Variations in compliance among hypertensive patients by drug class: Implications for healthcare costs.** *Clin Ther* 1997;**19**(6):1446-57.

[19] Kopjar B, Sales AE, Piñeros SL, *et al:* **Adherence with statin therapy in secondary prevention of coronary heart disease in Veterans Administration male population.** *Am J Cardiol* 2003;**92**(9):1106-8.

[20] Simpson E, Beck C, Richard H, *et al:* **Drug prescriptions after acute myocardial infarction: Dosage, compliance, and persistence.** *Am Heart J* 2003;145(3):438-44.

[21] Osterberg L, Blaschke T: **Adherence to medication.** *N Eng J Med* 2005;**353**(5):487-97.
